# EhVps23: A Component of ESCRT-I That Participates in Vesicular Trafficking and Phagocytosis of *Entamoeba histolytica*

**DOI:** 10.3389/fcimb.2021.770759

**Published:** 2021-10-29

**Authors:** Ausencio Galindo, Rosario Javier-Reyna, Guillermina García-Rivera, Cecilia Bañuelos, Sarita Montaño, Jaime Ortega-Lopez, Bibiana Chávez-Munguía, Lizbeth Salazar-Villatoro, Esther Orozco

**Affiliations:** ^1^ Departamento de Infectómica y Patogénesis Molecular, Centro de Investigación y de Estudios Avanzados del IPN, Ciudad de México, Mexico; ^2^ Programa Transdisciplinario en Desarrollo Científico y Tecnológico para la Sociedad, Centro de Investigación y de Estudios Avanzados del IPN, Ciudad de México, Mexico; ^3^ Laboratorio de Bioinformática y Simulación Molecular, Facultad de Ciencias Químico-Biológicas, Universidad Autónoma de Sinaloa, Sinaloa, Mexico; ^4^ Departamento de Biotecnología y Bioingeniería, Centro de Investigación y de Estudios Avanzados del IPN, Ciudad de México, Mexico

**Keywords:** vesicular trafficking, *Entamoeba histolytica*, ESCRT, phagocytosis, EhVps23

## Abstract

The endosomal sorting complex required for transport (ESCRT) is formed by ESCRT-0, ESCRT-I, ESCRT-II, ESCRT-III complexes, and accessory proteins. It conducts vesicular trafficking in eukaryotes through the formation of vesicles and membrane fission and fusion events. The trophozoites of *Entamoeba histolytica*, the protozoan responsible for human amoebiasis, presents an active membrane movement in basal state that increases during phagocytosis and tissue invasion. ESCRT-III complex has a pivotal role during these events, but ESCRT-0, ESCRT-I and ESCRT-II have been poorly studied. Here, we unveiled the *E. histolytica* ESCRT-I complex and its implication in vesicular trafficking and phagocytosis, as well as the molecular relationships with other phagocytosis-involved molecules. We found a gene encoding for a putative EhVps23 protein with the ubiquitin-binding and Vps23 core domains. In basal state, it was in the plasma membrane, cytoplasmic vesicles and multivesicular bodies, whereas during phagocytosis it was extensively ubiquitinated and detected in phagosomes and connected vesicles. Docking analysis, immunoprecipitation assays and microscopy studies evidenced its interaction with EhUbiquitin, EhADH, EhVps32 proteins, and the lysobisphosphatidic acid phospholipid. The knocking down of the *Ehvps23* gene resulted in lower rates of phagocytosis. Our results disclosed the concert of finely regulated molecules and vesicular structures participating in vesicular trafficking-related events with a pivotal role of EhVps23.

## Introduction

The plasma and internal membranes of the trophozoites of *Entamoeba histolytica*, the protozoan responsible of human amoebiasis, (WHO, 1997), are constantly producing invaginations, forming vesicles that fuse and split, and generating endosomes, multivesicular bodies (MVBs), tubes and pseudopodia; events necessary to capture, ingest and digest the prey, as well as for the selection of proteins that will be recycled or secreted. The incessant internal movements and the avid phagocytosis of the parasite are linked to its ability to invade tissues. Several molecules have been already identified as involved in these tasks (Bolaños et al., 2016; Castellanos-Castro et al., 2016a), but the molecular events underlying them are not well undersood.

The endosomal sorting complex required for transport (ESCRT), formed by the ESCRT-0, ESCRT-I, ESCRT-II, ESCRT-III complexes, and accessory proteins, is one of the main players in vesicle trafficking. Proteins that conform these complexes differ in sequence along organisms through the evolutionary scale (Leung et al., 2008), but conserve their functional domains that allow them to participate in cell division, endocytosis, virus budding and other functions (Calistri et al., 2021). In *E. histolytica*, ESCRT machinery is involved in phagocytosis (Lopez-Reyes et al., 2010; Avalos-Padilla et al., 2015; Avalos-Padilla et al., 2018), a virulence landmark of the parasite (García-Rivera et al., 1999). Thus, this protozoan is an excellent model to study the ESCRT machinery and its role in events involving vesicular trafficking, such as phagocytosis and tissue invasion (Alfred and Vaccari, 2016; Liu et al., 2019; Vietri et al., 2020).

At least 15 out of 20 ESCRT genes detected in *E. histolytica* are transcribed (Lopez-Reyes et al., 2010), and four of them, and the *Ehadh* gene, whose product is an associated protein of the ESCRT machinery (Bañuelos et al., 2012), appeared down- or up-regulated during phagocytosis. EhADH, an ALIX family protein involved in adhesion, interacts with EhVps32, a member of ESCRT-III, during MVBs formation (Bañuelos et al., 2012). In yeast, the Bro1 protein, an EhADH orthologue, forms a bridge between ESCRT-I and ESCRT-III complexes (Strack et al., 2003; Nikko and André, 2007).

The assembly sequence of *E. histolytica* ESCRT-III proteins on the endosomal membrane is essential for intraluminal vesicles (ILVs) formation (Avalos-Padilla et al., 2018). The active EhVps20 binds first to membranes and interacts with the active EhVps32, causing invaginations. EhVps32 recruits EhVps24 that allows the detachment of nascent vesicles inside the MVBs, whereas EhVps2 modulates the ILVs size (Avalos-Padilla et al., 2018). Then, the EhVps4-ATPase disrupts the complex to start a new assembly round (Lopez-Reyes et al., 2010). Here, we analyzed the ESCRT-I complex, particularly the EhVps23 protein, whose orthologue, TSG101, has been linked to malignant transformation in mammalian cells. In trophozoites in the basal state, EhVps23, was found in the cytoplasm, in MVBs and in the inner plasma membrane, but immediately after sensing the presence of RBCs, it moves to the attachment area of the prey. During this process, EhVps23 interacts with the EhADH, EhVps32 proteins, and the lysobisphosphatidic acid (LBPA). The knock-down of the *Ehvps23* gene resulted in a lower rate of phagocytosis, strengthening the role of EhVps23 in the virulence of the parasite.

## Materials and Methods

### Identification of EhVps23 and Phylogenetic Trees Construction

*H. sapiens* (TGS101) and *S. cerevisiae* Vps23 proteins sequences (access numbers: Q99816 and P25604, respectively), retrieved from the Uniprot database (https://www.uniprot.org) were used as query to search an *E. histolytica* Vps23 protein (EhVps23) in the AmoebaDB database (https://amoebadb.org/amoeba/app). Structural domains of the candidates were identified using the SMART genomics server (http://smart.embl-heidelberg.de/) and the motif tool of KEGG (https://www.genome.jp/kegg/).The predicted amino acid sequences of the putative EhVps23 (C4M9S4) were aligned with orthologous sequences. Data were submitted to phylogenetic analysis by UPGMA using the MEGA 5.05 software (Tamura et al., 2011). Bootstrapping was performed for 1000 replicates.

### 3D Structure Modeling

To obtain the EhVps23 3D model, we used the crystal structure of the Vps23 protein from *S. cerevisiae* (3R3Q:A) as a template on the I-TASSER server (https://zhanglab.dcmb.med.umich.edu/I-TASSER/) (Roy et al., 2010). After selecting the most energetically stable model, we evaluated their quality by the RAMPAGE server (http://mordred.bioc.cam.ac.uk/~rapper/rampage.php). The amino acid sequences of HsTSG101 were analyzed using the RaptorX server (http://raptorx.uchicago.edu/) to obtain the HsTSG101 3D structures (Källberg et al., 2012). The structures were visualized using the UCSF Chimera software. Similitudes beteween the UEV and Vps23 core domains of both proteins was analyzed using the RaptorX Structure Alignment tool.

### *E. histolytica* Cultures

*E. histolytica* trophozoites, strain HM1:IMSS, were axenically grown at 37°C in TYI‐S‐33 medium (Diamond et al., 1978) and harvested at logarithmic growth phase. To perform the experiments, the culture flasks were chilled at 4°C and trophozoites were collected by centrifugation. All experiments were performed at least three times by duplicate.

### Antibodies

As primary antibodies we used: mouse monoclonal α-histidine (α-His; Roche), mouse monoclonal α-BtUb (α-Ub; SantaCruz), rabbit α-EhADH (α-EhADH18) (Díaz-Hernández et al., 2021), mouse α-EhVps32 (α-EhVps32) (Avalos-Padilla et al., 2015), mouse α-LBPA (α-LBPA; Echelon Bioscience) and mouse monoclonal α-human actin (α-actin) (kindly given by Dr. Manuel Hernández, Cell Biology Department, CINVESTAV IPN). Secondary antibodies were: HRP-labelled α-rabbit, mouse and rat IgGs (Zymed) for western blot. FITC-labelled α-rabbit and α-mouse IgGs and TRITC-labelled α-rat IgGs (Life Technologies) for immunofluorescence. For immunoelectron microscopy experiments, we used α-rat IgG, conjugated with 10 nm gold particles and α-mouse IgG, conjugated with 20 nm gold particles (TED Pella Inc).

### PCR Assays

Total RNA was isolated from trophozoites using TRIzol reagent (Invitrogen), according to manufacturer’s recommendations. Complementary DNA (cDNA) was synthesized using oligo dT primers and the Superscript II reverse transcriptase (Invitrogen). PCR amplifications were carried out using Q5 ^®^ High-Fidelity DNA Polymerase (Biolabs) with 20 ng of cDNA as templates, and specific primers. Products were separated by electrophoresis in 1% agarose gels, and stained with ethidium bromide. As controls for PCR amplifications, instead of DNA, nuclease-free water was used.

### Plasmid Constructs

The *Ehvps23_1123-1485 bp_* or *Ehvps23_1-341 pb_* sequences were PCR-amplified using cDNA as template and specific primers: for *Ehvps23_1123-1485 bp_*: sense 5′-GGGGTACCATGGAAGAGTCTGAAGAAATACTTCATG-3′, antisense 5′-CCGGATCCTTATTCAGTTATGCAATACTTTGCATGAA-3′; for *Ehvps23_1-341 bp_* 5´-GGGAGCTCATGCAACCTATAAACAATGAAAAGAATATTAAC-3´ and the antisense 5′- CCGGTACCAACTTTCCTAATAACACCATTTTCATCTAC-3. Underlined sequences correspond to the enzyme restriction sites added to the primers. Fragments were cloned in pColdI for producing a recombinant protein tagged with 6X His, and in pL4440 for silencing experiments, generating the *pColdIEhvps23_1123-1485 pb_* and *pL4440Ehvps23_1-341 pb_*, plasmids, respectively. The quality of the constructs were verified by restriction enzyme analyses and automatic DNA sequencing.

### Expression and Purification of Recombinant His-EhVps23 Protein

*E. coli* BL21 (DE3) bacteria were transformed with the *pColdIEhvps23_1123-1485 bp_* plasmid to produce the His-tagged EhVps23_375-494 aa_ recombinant protein (rEhVps23). Protein expression was induced by 0.4 mM isopropyl beta-D-thiogalacto pyranoside (IPTG) in the LB medium for 16 h at 16°C. After lysis, the rEhVps23 protein was recovered from the inclusion bodies, using the solubilization buffer containing 20 mM Tris-HCL pH 7.5; 8 M Urea, 0.5 M NaCl, 1 mM β-mercaptoehtanol and 5 mM imidazole. rEhVps23 protein was purified through chromatography in Ni-Sepharose 6 columns (GE Healthcare in the NGC Q Chromatographic System, Bio-Rad) (de la Cruz et al., 2019). Identity and integrity of the purified rEhVps32 protein was verified by 10% SDS-PAGE and western blot assays.

### Generation of Polyclonal Antibodies Against EhVps23

150 μg of purified rEhVps23 protein were emulsified in Titer-Max Gold adjuvant (1:1) (Sigma) and subcutaneously and intramuscularly inoculated in Wistar rats (Avalos-Padilla et al., 2015). One more dose without Titer-Max was injected after 4 weeks. Pre-immune sera were obtained before immunizations.

### Western Blot Assays

Total extracts from trophozoites (prepared in the presence of 100 mM PHMB, 2.7 mM E64 and inhibitors cocktail) and bacterial lysates (prepared in the presence of 100 mM PMSF), were separated by SDS-PAGE gels, transferred onto PVDF or nitrocellulose membranes, and probed with rat α-EhVps23 (1:500), mouse α-His (1:500), mouse α-Ub (1:100), rabbit α-EhADH18, (1:500), mouse α-EhVps32 (1:500) or mouse or α-human actin (1:3,500) antibodies. Membranes were incubated with the species-specific horseradish peroxidase (HRP)-labelled secondary antibodies (1:10,000), and developed with the ECL Prime detection reagent (GE-Healthcare). Pre-immune serum was used as a control and competition experiments were performed using the recombinant protein to assure the proper identification of EhVps32 in trophozoites lysates.

### Phagocytosis Assays

For phagocytosis assays the trophozoites were incubated for 0, 2, 5, 15, and 30 min with RBCs (1:25) at 37°C. At different times, trophozoites were prepared for immunofluorescence (García-Rivera et al., 1982) and observed through the laser confocal microscope. Other preparations were stained by Novikoff technique (Novikoff et al., 1972) and ingested erythrocytes were counted in 100 trophozoites through the light microscope (Axiolab, Zeiss). For pulse-chase experiments, incubation with RBCs was carried out for 2 min at 37°C. Then, preparations were incubated with TYI-water (2:1) for 5 min at 37°C to remove the adhered and non-ingested erythrocytes, cell mixtures were again incubated at 37°C for different times and samples were treated for immunofluorescence and observed through the Carl Zeiss LMS 700 laser confocal microscope.

### Immunofluorescence Assays

Trophozoites (grown on coverslips) were fixed with 4% paraformaldehyde at 37°C for 1 h, permeabilized with 0.2% Triton X‐100 and blocked with 10% fetal bovine serum in PBS. Preparations were incubated at 4°C overnight (ON) with α-EhADH18 (1:50), or α-EhVps32 (1:50), α-LBPA (1:30), or α-EhVps23 (1:50) or α-Ub (1:15) antibodies, followed by incubation for 30 min at 37°C with TRITC-labelled α-rat IgG for α-EhVps23, FITC-labelled α-rabbit IgG for α-EhADH18 and α-EhVps32, or FITC-labeled α-mouse IgG for α-LBPA and α-Ub (1:100). Preparations were preserved using Vectashield antifade reagent (Vector) and then, 0.5 µm laser sections were examined through the confocal microscope and processed with ZEN 2009 Light Edition Software (Zeiss). To evaluate the co-localization between molecules, Pearson coefficients were obtained from at least 25 confocal images using the ImageJ 1.45v software and the JACoP plugin.

### Immunoelectron Microscopy

Samples were prepared for TEM as described (Bolaños et al., 2016). Briefly, trophozoites were fixed with 4% paraformaldehyde and 0.5% glutaraldehyde in PBS for 1 h at room temperature. Samples were embedded in LR White resin (London Resin Co) and polymerized under UV at 4°C for 48 h to obtain thin sections (60 nm) that were mounted on Formvar‐covered nickel grids followed by ON incubation with α‐EhVps23 (1:30) and α-LBPA (1:10). The thin sections were incubated ON with secondary antibodies (1:50) conjugated to 10‐nm (for α‐EhVps23) or 20-nm (for α-LBPA) gold particles, contrasted with uranyl acetate and lead citrate and observed through a Joel JEM‐1011 transmission electron microscope.

### Protein-Protein Docking

The 3D predicted and refined structures of EhADH and EhVps32 proteins were used for docking experiments (Montaño et al., 2017) whereas the 3D structure of EhVps23 protein was generated in this work. The snapshots were obtained using the clustering analysis in the last 50 ns of the MDS with the Carma software (Koukos and Glykos, 2013). The protein-protein docking analysis were done employing different conformers with the Cluspro server (Comeau et al., 2004a; Comeau et al., 2004b; Kozakov et al., 2013). The conformers with the highest cluster members and the lowest energy, calculated in FireDock (Mashiach et al., 2008), were taken for analysis on the PDBSum server (Laskowski et al., 1997). Visualization of 3D structures was performed by VMD (Humphrey et al., 1996). To obtain the binding site between EhVps23 and EhUbiquitin, the crystal structure of EhUbiquitin (PDB:4GSW: B) and Vps23 3D structure from yeast were structurally aligned to get the coordinates of the binding site between the EhVps23 with the EhUbiquitin.

### Protein-Ligand Docking

The LBPA structure was obtained and optimized as reported (Castellanos-Castro et al., 2016b). Molecular docking was performed using Autodock4 (Morris et al., 2009), Autodock Vina (Trott and Olson, 2009) and AutoDock Tools 1.5.7, and performed using a grid box of 80 Å and a grid space of 0.375 Å. Then, polar hydrogen atoms and Kollman charges (Singh and Kollman, 1984) were localized in EhVps23. For scoring sampling, the Lamarckian Genetic Algorithm using an initial randomized population of 100 individuals and a maximum number of energy evaluations of 1 x 10^7^ runs were performed. The predicted lowest energy binding position was considered for the analysis.

### Immunoprecipitation Assays

Trophozoites were lysed in the presence of 10 mM Tris‐HCl, 50 mM NaCl, and proteases inhibitors, by cycles of freeze‐thawing in liquid nitrogen and vortexing. Immunoprecipitation assays were performed using 200 μl of protein G‐agarose (Invitrogen) and α-EhVps23 antibody. Immunoprecipitated proteins were analyzed by western blot assays using α-EhVps23, α-EhADH18 α-EhVps32, and α-Ub, as described above.

### *Ehvps23* Gene Silencing Based on dsRNA

To knock-down the *Ehvps23* gene we used bacterial double-stranded RNA (dsRNA) and parasite soaking experiments (Solis et al., 2009). Briefly, the competent RNase III-deficient *E. coli* strain HT115 (rnc14:DTn10) was transformed with the *pL4440Ehvps23_1-341 pb_*. Bacteria were grown at 37°C in LB or 2YT broth for dsRNA expression, in the presence of ampicillin (100 mg/ml) and tetracycline (10 mg/ml), using 2 mM (IPTG) for induction, ON at 37°C. Then, the bacterial pellet was mixed with 1 M ammonium acetate and 10 mM EDTA, incubated with phenol:chloroform:isoamyl alcohol (25: 24:1) and centrifuged. The supernatant was mixed with isopropanol, centrifuged, and the nucleic acid pellet was washed with 70% ethanol. DNase I (Invitrogen) and RNase A (Ambion) were added to eliminate ssRNA and dsDNA molecules; *Ehvps23*-dsRNA was washed again with isopropanol and ethanol, analyzed by agarose gel electrophoresis and concentration was determined by spectrophotometry. Purified *Ehvps23*-dsRNA (5 μg/ml) were added to the trophozoites (3.0 × 10^4^) growing in TYI-S-33 complete medium tand incubated at 37°C for 72 hr. Cells growing in standard conditions (without dsRNA, or with an unrelated dsRNA) were used as controls.

### Statistical Analyses

Values for all experiments were expressed as the mean and standard error of at least three independent assays, carried out by duplicate. Statistical analyses were performed using the GraphPad Prism v5.01 software by a paired Student’s t test. *p< 0.05; **p< 0.01, and ***p< 0.001.

### Ethics Statement

CINVESTAV fulfills the standard of the Mexican Official Norm (NOM-062-ZOO-1999) “Technical Specifications for the Care and Use of Laboratory Animals”, based on the Guide for the Care and Use of Laboratory Animals (“The Guide,” 2011, NRC, USA with the Federal Register Number BOO.02.03.02.01.908), awarded by the National Service for Agrifood Health, Safety and Quality (SENASICA). This organization verifies the state of compliance of such NOM in Mexico and belongs to the Ministry of Agriculture and Rural Development. The Institutional Committee for Animal Care and Use (IACUC/Ethics committee) from CINVESTAV, the regulatory office for research protocols approval involving the use of laboratory animals, reviewed, and approved all animal experiments (Protocol Number 0505-12, CICUAL 001).

## Results

### EhVps23 Has Functional Domains With Similarities to Putative Orthologues

**Figure 1:**
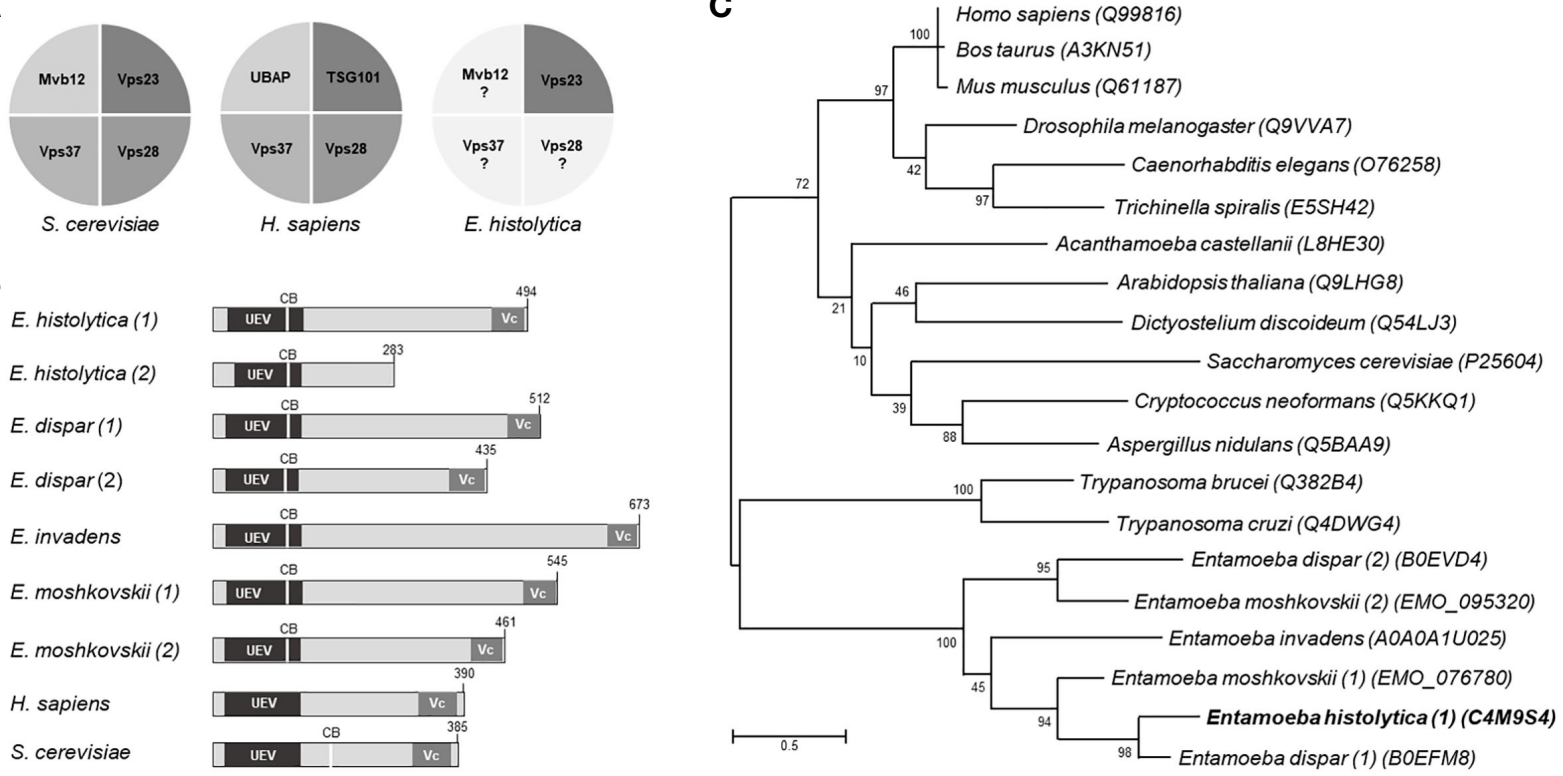
Structural domains and phylogenetic trees of Vps23 proteins. **(A)** Proteins that form the ESCRT-I complex in *Saccharomyces cerevisiae*, *Homo sapiens* and *Entamoeba histolytica*. **(B)** Schematic comparison of the Vps23 domains of *Entamoeba spp*, *H. sapiens* and *S. cerevisiae*. UEV, ubiquitin binding domain; Vc, Vps23 core domain; CB, Clathrin box. Numbers at the right correspond to the number of amino acids forming the proteins. **(C)** Phylogenetic tree indicating the position of *E histolytica* Vps23 (EhVps23) protein among different species. Numbers on horizontal lines indicate the confidence percentages of the tree topology from bootstrap analysis of 1,000 replicates.

In humans and yeast, ESCRT-I is an elongated heterotetrameric complex (1:1:1:1) that interacts by one side with the HRS/STAM (ESCRT-0), and by the other with the Vps36 protein (ESCRT-II). It is formed by TSG101 (named Vps23 in yeast), Vps28, Vps37, and UBAP1 (MVB12 in yeast) proteins (**Figure 1A**). Vps23 from *Saccharomyces cerevisiae* and *Homo sapiens* orthologues have been already crystallized and their role in vital cellular functions has been studied (Kostelansky et al., 2007; Im et al., 2010; Ren and Hurley, 2011; Flower et al., 2020). In *E. histolytica*, the ESCRT-III proteins have been studied, but little is known on the other complexes of the ESCRT machinery. Using as templates the Vps23 core and the UEV (referred to the ubiquitin E2 variant domain found in TSG101 protein and in many other ubiquitin-binding proteins) domains of the *S. cerevisiae* Vps23 protein, we uncovered two contigs in the *E. histolytica* genome with intronless open reading frames, predicting 494 and 283 amino acid sequences (**Figure 1B**), but the smaller one (Leung et al., 2008), lacks the Vps23 core domain, that is the Vps23 proteins signature, thus, it was not considered for this work. Additionally, we did not detect consensus sequences with domains described for Vps28, Vps37, and MVB12 (Kostelansky et al., 2006; Kostelansky et al., 2007). Lopez-Reyes (Lopez-Reyes et al., 2010) found a Vps37D protein, but its tertiary structure lacks the alpha helixes necessary for the EhVps23 functions (Kostelansky et al., 2007). However, given the variation of the ESCRT proteins of *E. histolytica*, compared with their orthologues (Lopez-Reyes et al., 2010; Avalos-Padilla et al., 2018) (**Figures S1, S2**), the existence of other ESCRT-I members cannot be discarded.

The phylogenetic analysis showed two genes in *E. dispar*, one in *E. invadens* and two in *E. moshkovskii* grouped in a clade close to *Trypanosoma cruzi* and *T. brucei*, in which *E. histolytica* is between *E. dispar* and *E. moshkovskii* (**Figure 1C**).

### The Main Domains of EhVps23 3D Structure Overlap With TSG101

**Figure 2:**
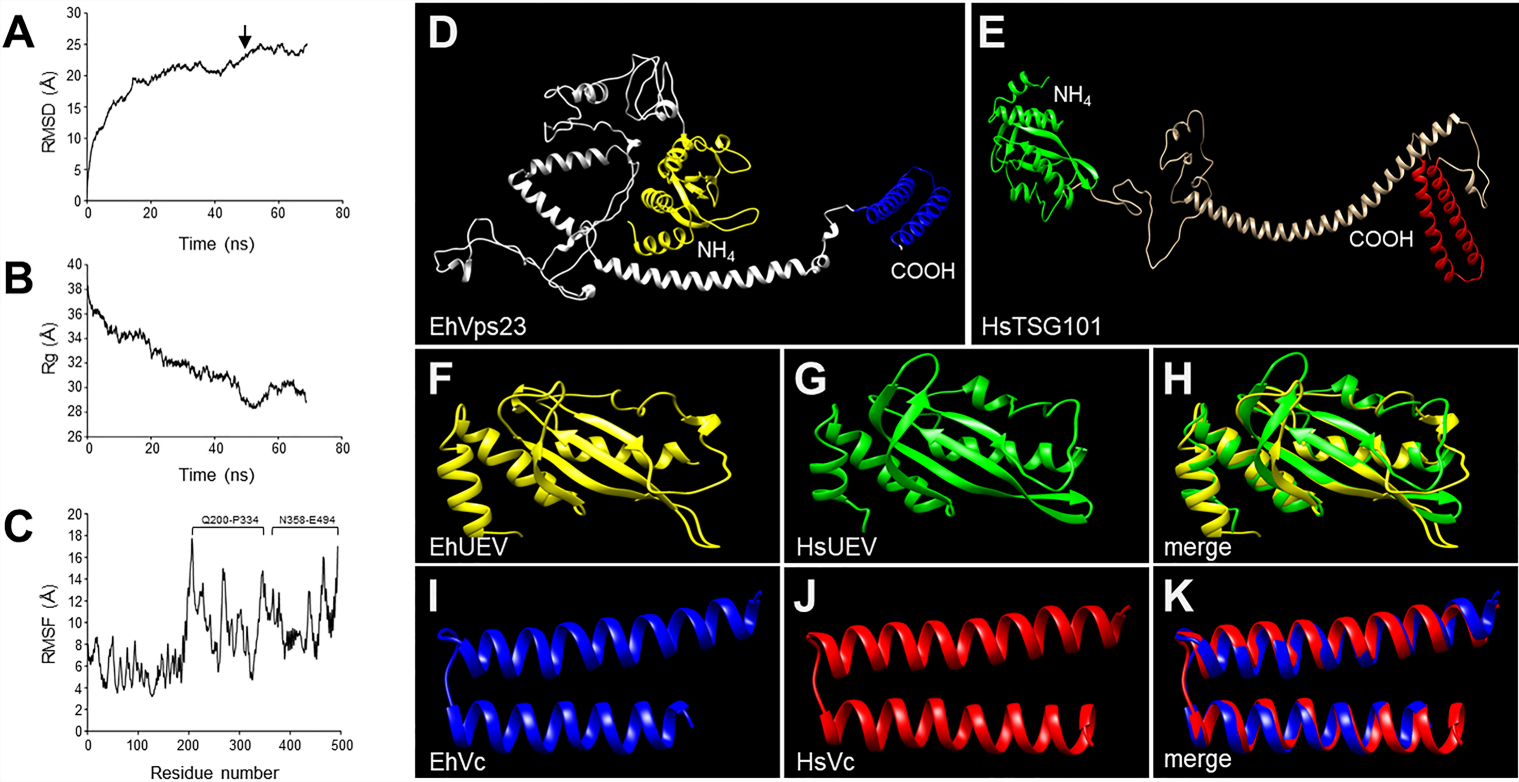
MDS of EhVps23 and its predicted 3D model. **(A)** RMSD: Root mean square deviation. Arrow: signals the ns of protein stability. **(B)** Rg: Radius of gyration. **(C)** RMSF, Root mean square fluctuation. Brackets: The most flexible regions. **(D)** Prediction of the EhVps23 tertiary structure. **(E)** HsTSG101 tertiary structure. **(F)** UEV domain of the EhVps23 protein [21 to 140 amino acids]. **(G)** UEV domain of HsTSG101 [17 to 141 amino acids]. **(H)** Overlapping of EhVps23 and HsTSG101 UEV domains. **(I)** Vps23 core (Vc) domain of EhVps23 [436 to 492 amino acids] **(J)** Vps23 core of HsTSG101 [322 to 381 amino acids]. **(K)** Overlapping of EhVps23 and HsTSG101 Vc domains.

The 3D structure of EhVps23 was obtained from the I-TASSER server. It was selected according to its C-score and the best Ramachandran plot values after MDS during 70 ns in a soluble environment (Figures 2A–D). The Ramachandran plot (https://zlab.umassmed.edu/bu/rama/) (Hooft et al., 1997) showed 76.2% amino acids in the favored regions, 97.3% in the allowed regions, and 2.6% in the outlier ones (see **Figure S-3**), suggesting that the refinement of torsion angles of certain amino acids through MDS, occurred. RMSD calculations indicated that EhVps23 reached the equilibrium after 50 ns (**Figure 2A**), and Rg data revealed that EhVps23 is an extended protein that compacts through the dynamics (**Figure 2B**). The RMSF values showed a peak of fluctuation in a disordered region from Q200 to P334 amino acids, formed by random coils. Another fluctuation peak was located at the carboxy-terminus from N358 to E494 amino acids, where an α-helix is formed (**Figure 2C**). Thus, these analysis showed that EhVps23 has the 3D structure described for its orthologues (Kostelansky et al., 2007; Im et al., 2010; Ren and Hurley, 2011; Flower et al., 2020) with fourteen alpha helices and six beta-sheets (**Figure 2D**).

The UEV (**Figures 2F, G**) and Vps23 core (**Figures 2I, J**) domains of EhVps23 (**Figure 2D**) and human TSG101 (**Figure 2E**) exhibited 82% (RMSD: 2.44) and 90% (RMSD: 0.84) structural identity, respectively (**Figures 2H–K**). Besides, the carboxy terminus (Vps23 core domain) of EhVps23 presents the alpha helixes described as responsible for the ESCRT-I heterotetramer formation, suggesting that these structures could contact amoebic proteins that we did not identify here. The analysis of the 3D structures strongly suggests that EhVps23 is indeed an orthologue of yeast Vps23 and human TSG101 proteins.

### In Trophozoites in Basal State, EhVps23 Is Localized in Vesicles

**Figure 3:**
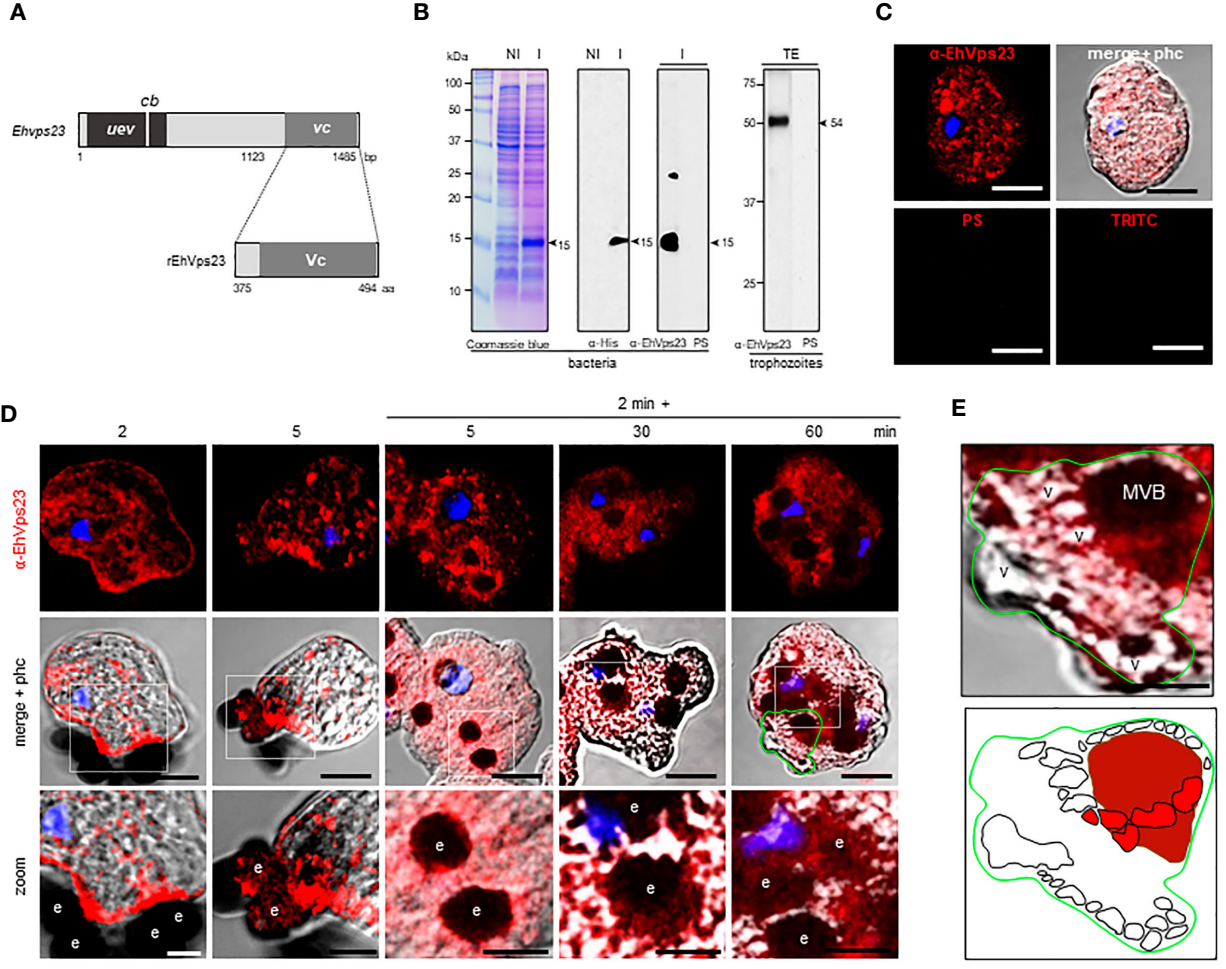
Localization of EhVps23 in the trophozoites. **(A)** Schematic representation of the *Ehvps23* gene and the fragment cloned to express the rEhVps23 polypeptide (375 to 494 amino acids) used to generate the rat α-EhVps23 antibodies. **(B)** Coomassie blue-stained gels (10% PAGE-SDS) containing protein extracts of non-induced (NI) and induced **(I)** bacteria. At right: western blot assays of induced bacteria proteins **(I)** or total extracts (TE) of trophozoites developed with α-His tag or α-EhVps23 antibodies. PS; pre-immune serum. Numbers at left: molecular weight standards. **(C)** Confocal images of trophozoites in basal conditions incubated with the α-EhVps23 antibodies and TRITC-labelled α-rat secondary antibodies, or with PS or only with the secondary antibody (TRITC). **(D)** Confocal microscopy images were obtained from trophozoites incubated with RBCs for 2 and 5 min at 37°C; or with trophozoites incubated for 2 min at 37°C with the RBCs, the adhered and not ingested RBCs were removed by osmotic shock and preparations were incubated again at 37°C for 5, 30 and 60 min (2 min + 5, 30 and 60 min). Lower panel: Zoom of regions marked by white squares. **(E)** Magnification of MVBs surrounded by multiple vesicular structures. Lower panel: Schematic depiction of **(E)**. v, vesicles; MVBs, multivesicular bodies. Scale bar = 10 μm.

To study the location and follow the movement of EhVps23 in the trophozoites, we produced specific antibodies against the carboxy terminus of the protein. A fragment containing 1123 to 1485 bp of the gene, absent in the truncated contig (**Figure 3A**), was used to obtain the recombinant EhVps23_375-494 aa_ polypeptide (rEhVps23) in *Escherichia coli*, labelled with a histidine tag (His-tag). The polypeptide was injected into rats to generate antibodies. In western blot assays, antibodies recognized a 15 kDa band (**Figure 3B**). The rat α-EhVps23_375-494 aa_ antibodies (α-EhVps23) (**Figure 3B**) reacted with the rEhVps23 polypeptide in bacteria and revealed a 54 kDa band in trophozoites lysates (**Figure 3B**), the molecular weight deduced from the amino acids sequence. Through the confocal microscope, these antibodies detected EhVps23 clustered, probably in vesicles (**Figure 3C**).

### Under the Erythrocyte’s Stimulus, EhVps23 Moves to the Adherence Spots and Surrounds the Ingested RBCs

To explore the fate of EhVps23 under the stimulus of phagocytosis, the protein was tracked using the α-EhVps23 and TRITC-labelled anti-rat secondary antibodies (**Figure 3D**). Confocal images of trophozoites in contact with red blood cells (RBCs) revealed the displacement of EhVps23 at the site of contact with the target cell. At 2 and 5 min, the label was localized in the adhered and ingested RBCs (**Figure 3D**, 2 and 5 min). In other experiments, the cell mixture was incubated at 37°C for 2 min, and adhered and non-ingested RBCs were removed by mild osmotic shock to avoid the noise of new ingested cells, and trophozoites were again incubated at 37°C. In these experiments, at 5 min (2 + 5), the antibodies detected larger vesicles in the cytoplasm and around the ingested erythrocytes. At 2 + 30 and 2 + 60 min, some of the protein appeared in cytoplasm vesicles not in contact with the ingested RBCs, whereas another part of the label remained surrounding the RBCs (**Figure 3D**). Elongated and round vesicles were observed close to or inside of large phagosomes and MVBs (**Figures 3D, E**). Magnification of the images revealed chains of relatively ordered vesicles of different sizes, as if they were forming specific cellular structures. Many of them, seem to be emerging from or fusing to other vesicles, however other type of experiments are needed to prove this (**Figure 3E**). In synthesis, the analysis of many confocal images revealed that, after the RBCs stimulus, EhVps23 moves from the cytoplasm to the adherence site at the plasma membrane, then, after ingestion, it borders the erythrocytes-containing phagosomes and MVBs; later, it appears close to or inside of vesicles, giving a panoramic of the movement of EhVps23 during the active vesicular trafficking and phagocytosis of trophozoites.

### Transmission Electron Microscopy Disclosed EhVps23 Located in MVBs, Plasma Membrane and Endosomes

**Figure 4:**
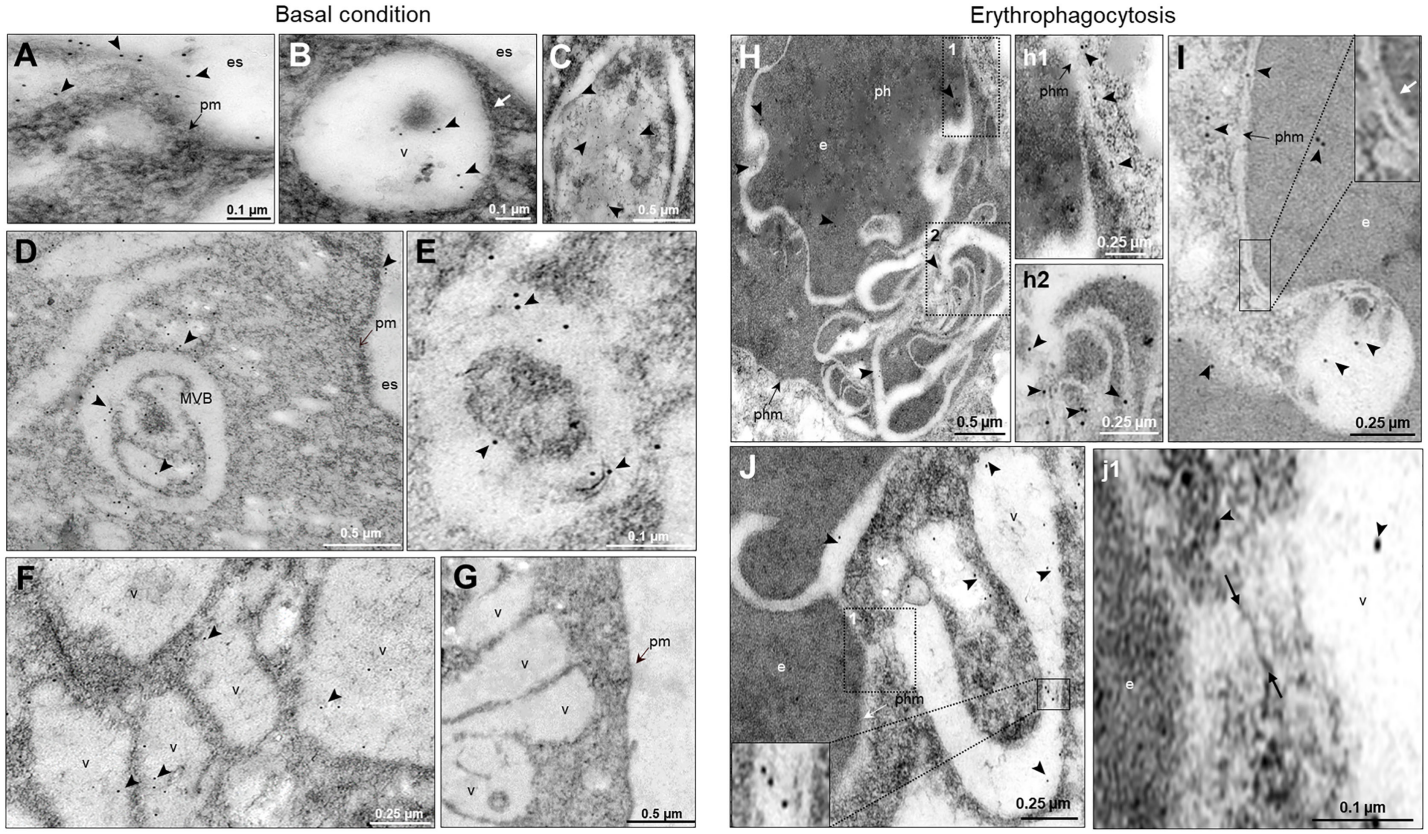
Location by TEM of the EhVps23 protein in trophozoites in basal conditions and during erythrophagocytosis. In basal condition: **(A)** Thin sections of trophozoites incubated with α‐EhVps23 and α‐rat secondary antibodies coupled to 10 nm colloidal gold particles. **(B)** Vesicles (v) close to the plasma membrane (pm) and in the extracellular space (es) containing labelled EhVps23. White arrows in B: ripples of the vesicular membrane. **(C–E)** Multivesicular bodies (MVB) and vesicles (v) labelled with EhVps23. **(F)** EhVps23 in vesicles (v). **(G)** Control trophozoites using only secondary antibodies. During erythropagocytosis: **(H)** Thin section showing phagosome (ph) with an erythrocyte (e) exhibiting convoluted membranous structures with labelled EhVps23, phm: phagosome membrane. (h1) and (h2): Magnification of the black squared areas in H **(I)** Vesicle apparently entering the erythrocyte (e) inside a phagosome, where the two membranes can be observed together (inset). The white arrow is signaling the vesicle and phagosome membrane (phm). **(J)** Tubular and round vesicles containing EhVps23. (j1) Magnification of the discontinuous black square. Arrowheads: labelled EhVps23, white arrows mark the join between two vesicles. es, extracellular space; pm, plasma membrane; v, vesicles; phm, phagosome membrane; e, erythrocytes.

To obtain further data on the moving of EhVps23, we performed transmission electron microscopy (TEM) analysis using the α-EhVps23 and gold-labeled secondary antibodies. TEM images revealed the protein on the plasma membrane and in the extracellular space, close to several vesicles of about 0.6-0.9 µm, suggesting that EhVps23 could be secreted (**Figures 4A, B**) and inside MVBs and ILVs (**Figures 4C–E**), like the ones discovered in *Dictyostelium discoideum* treated with the drug U1866A (cationic sterol) that impairs fusion of endosomes with lysosomes, which causes the accumulation of membranous structures (Marchetti et al., 2004), and in *E. histolytica* overexpressing EhADH (Bañuelos et al., 2012). These vesicles appeared surrounded by other labelled vesicles (**Figures 4D, E**), like those observed through the confocal microscope. The different numbers and sizes of the vesicles could suggest a distinct maturation state of MVBs. Around some MVBs and vesicles, there were labelled networks (**Figure 4B, E**). Scarce label was detected free in the cytoplasm (**Figures 4A–F**), and it did not appear in controls treated with secondary antibodies (**Figure 4G**).

After 15 min contact with RBCs, TEM images showed convoluted membranous structures penetrating the phagosomes and ingested RBCs, and the protein was detected there (**Figures 4H, J**). Tubular structures appeared very close each other (**Figures 4I, J**). This and the white convoluted arrangements inside the RBCs suggest that some vesicles could be transporting different molecules including lytic enzymes, (necessary for digestion of the cargo) from one vesicle to another and that possibly their content are shed on the RBCs-containing phagosomes. The digested hemoglobin could account for the form and size of the arrangements observed in MVBs or phagosomes with ingested RBCs. Like in basal conditions, the labelled phagosomes were surrounded by other labelled vesicles (**Figure 4J**). By their appearance and their expansion inside ingested RBCs, lytic enzymes and lipids could be transported there.

### EhVps23 Associates to Ubiquitin During Phagocytosis

**Figure 5:**
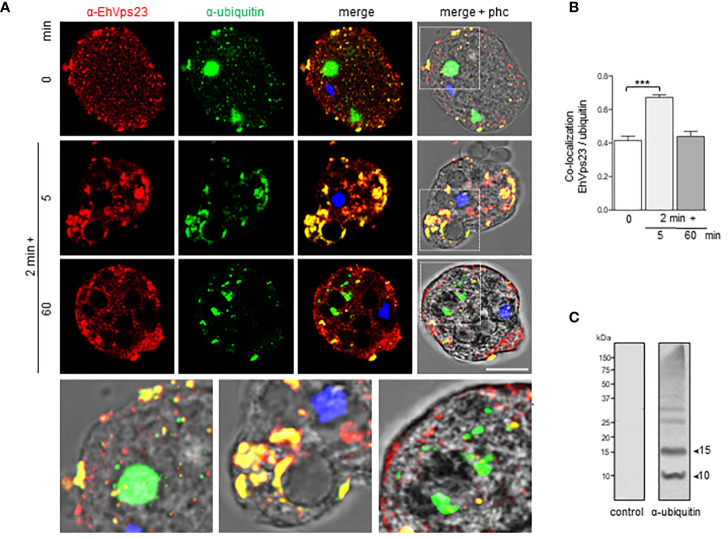
Co-location of EhVps23 and EhUbiquitin in trophozoites. Trophozoites were incubated for 2 min at 37°C with RBCs, non-ingested RBCs were lysed and preparation incubated again for different times. **(A)** Trophozoite in basal conditions (0 time) and during distinct times of phagocytosis, treated with α-EhVps23 (red), α-ubiquitin (green) antibodies, and DAPI (blue). Lower panel: Magnification of the regions marked with white squares in merge+phc. phc: phase contrast. Scale bar = 10 µm. **(B)** Pearson’s coefficient for EhVps23 and ubiquitin co‐localization (***) P < 0.001. **(C)** Western blot analysis of trophozoites lysates in basal condition, developed with the α-ubiquitin antibodies and control. Numbers at left: molecular weight standards.

According to Dupré et al., a yeast protein complex, containing the Vps23 protein, or its mammalian counterpart, TSG101, contains ubiquitin receptors (Dupré et al., 2001). *E. histolytica* possesses the ubiquitination machinery (Bosch and Siderovski, 2013), this, and the predicted ubiquitin domain in EhVps23 and its movement during phagocytosis (**Figure 5A**), suggested that EhVps23 could be also ubiquitinated or be in contact with ubiquitinated proteins. Thus, we used a commercial antibody against ubiquitin to explore this. In basal conditions, the α-ubiquitin antibodies detected ubiquitin in the cytoplasm of trophozoites, but reduced co-localization with EhVps23 was observed (**Figure 5A**). However, after 2 + 5 min of phagocytosis, an extensive co-localization of both proteins emerged, and after 2 + 60 min, when EhVps23 was returned to its basal state, both proteins were observed again separated (**Figures 5A, B**), suggesting that ubiquitination could activate EhVps23 to carry out its tasks during phagocytosis, which is supported by the fact that K50, K67, K100, K370, K403, K435, K436 and K445 amino acids are susceptible of ubiquitination. However, the presence of the UEV domain also makes probable that EhVps23 is interacting in a non-covalent way with ubiquitinated proteins, and that this interaction allows EhVps23 to perform its functions during erythrophagocytosis. Both events could be happening. After a certain time, ubiquitin and EhVps23 appeared again separated in the cell. In western blot assays, the α-ubiquitin commercial antibody recognized several bands (**Figure 5C**) corresponding to EhUbiquitin-conjugated proteins. The 10 and 15 kDa bands detected by the antibody exhibited the molecular weight deduced by the amino acids forming the *E. histolytica* ubiquitin (Wostmann et al., 1992; Arya et al., 2012).

### EhVps23 Co-Localizes With EhADH, EhVps32, and LBPA

EhADH participates along the process of phagocytosis (García-Rivera et al., 1999). Associated with the EhCP112 cysteine protease, it forms the EhCPADH complex involved in the adherence to and destruction of target cells (García-Rivera et al., 1999). After the EhCPADH complex contacts the prey, it surrounds the ingested RBCs and penetrates the phagosomes, interacting with ESCRT-III proteins during ILVs formation (Avalos-Padilla et al., 2018). EhADH also binds to cholesterol and to the EhNCP1 and EhNCP2 proteins, responsible for cholesterol transport (Bolaños et al., 2016), and to the LBPA phospholipid (Castellanos-Castro et al., 2016a), acting as a scaffold molecule, performing distinct cellular functions. We explore here the relationship of EhVps23 with EhADH, EhVps32 and LBPA, three molecules involved in phagocytosis.

**Figure 6:**
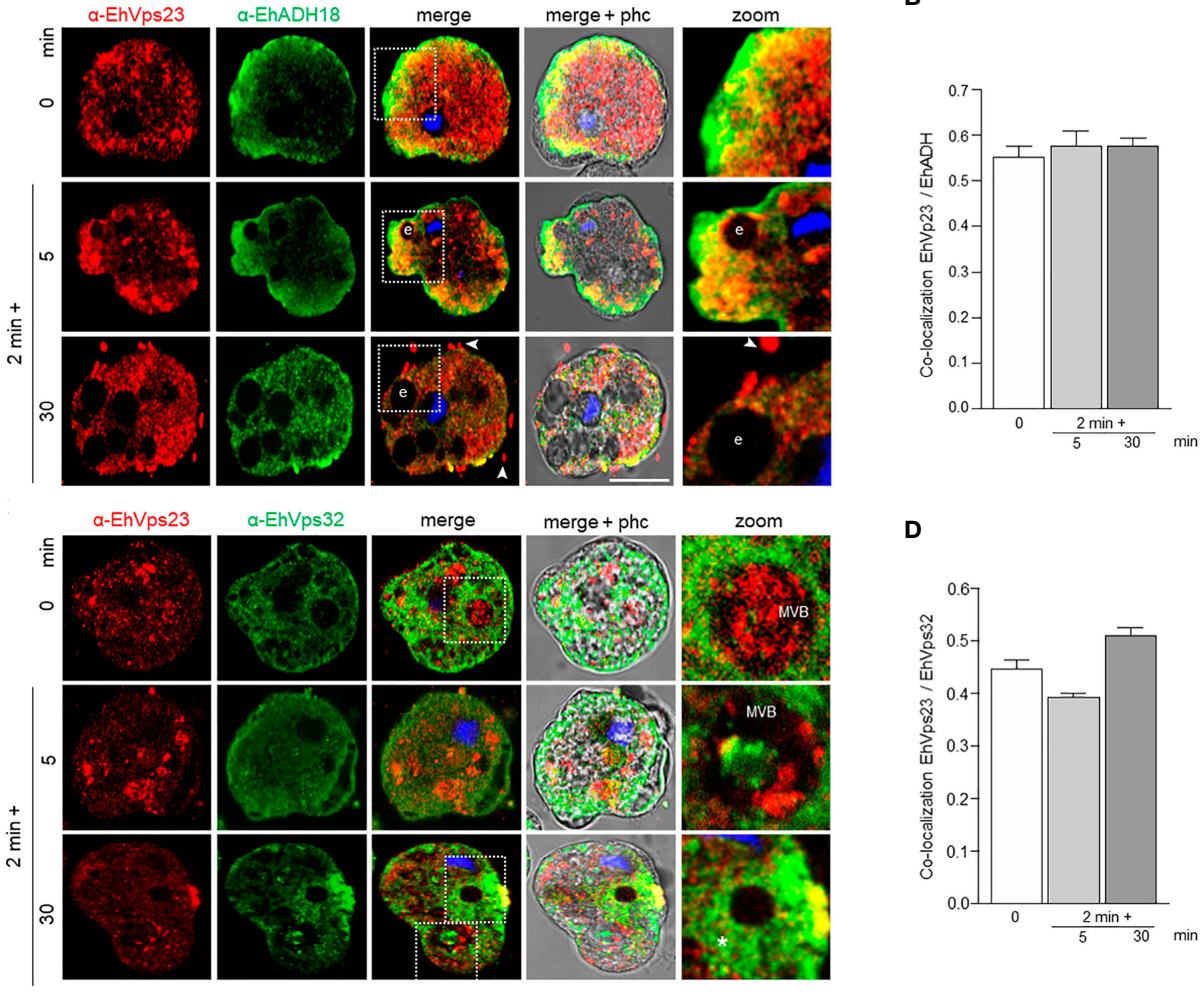
Co-localization of EhVps23 and EhADH or EhVps32 in trophozoites. Trophozoites were incubated for 2 min at 37°C with RBCs, and after lysis of non-ingested RBCs, incubated again for 5 and 30 min. **(A)** Confocal images of trophozoites treated with α-EhVps23 (red), α-EhADH18 (green) antibodies and DAPI (blue). **(B)** Pearson’s coefficient for EhVps23 and EhADH co-localization. **(C)** Confocal images of trophozoites treated with α-EhVps23 (red), α-EhVps32 (green) antibodies and DAPI (blue). **(D)** Pearson’s coefficient for EhVps23 and EhVps32 co-localization. Zoom: Magnification of regions marked by squares in merged images. Arrowheads: EhVps23 in the extracellular space. Asterisks: networks labelled by the α-EhVps32 antibody. e, erythrocytes; MVB, multivesicular bodies; phc, phase contrast. Scale bar = 10 μm.

In basal conditions, EhADH and EhVps23 co-localized in regions close to the inner plasma membrane, as well as in small vesicles (**Figures 6A, B**). After the trophozoites sense the RBCs, both proteins move to the phagocytic cups (**Figure 6A**). At longer times, interaction persisted in the plasma membrane. Interestingly, we distinguished EhVps23 forming bunches of vesicles close to the membrane and in some labelled spots outside the cell with EhADH (**Figure 6A**, 30 min). A recent proteomic study describes the presence of EhVps23 protein in secretion products (Sharma et al., 2020). The proximity and the cellular movement of these two proteins during phagocytosis support their involvement in vesicular traffick and phagocytosis. In contrast, we detected a minor interaction between EhVps23 and EhVps32 (**Figures 6C, D**). Interestingly, both proteins were distinguished inside MVBs, indicating a specific function of EhVps23 that could act as a carrier of molecules that are directed to these structures.

**Figure 7:**
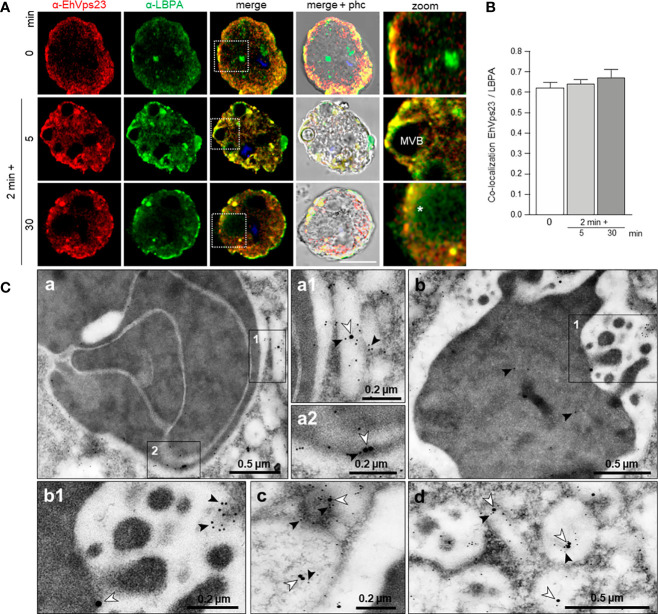
Co-localization of EhVps23 and LBPA in trophozoites. Trophozoites were incubated for 2 min at 37°C with RBCs and after removing the non-ingested RBCs, preparations were re-incubated for 5 and 30 min, and processed for immunofluorescence. **(A)** Confocal images of trophozoites treated with α-EhVps23 (red) and α-LBPA (green) antibodies, and DAPI (blue). Zoom: Magnification of regions marked by squares in merged images. Asterisks: network labeled with the α-LBPA antibody. MVB, multivesicular bodies; phc, phase contrast. Scale bar = 10 μm. **(B)** Pearson’s coefficient for EhVps23 and LBPA co-localization. **(C)** TEM localization of EhVps23 and LBPA in trophozoites after erythrophagocytosis. (a): Thin sections of trophozoites incubated with α-EhVps23 and α-LBPA antibodies followed by incubation with gold-labelled secondary antibodies of 10 and 20‐nm, respectively. (a1) and (a2): Magnification of the areas in black squares in (a). (b) Degraded RBC inside a phagosome labelled with EhVps23 and LBPA. (b1) Magnification of the black squared area in (b). (c) and (d): in vesicles of different sizes and shapes market with EhVps23 and LBPA. White arrowheads point out LBPA label, and black arrowheads EhVps23.

LBPA associates to EhADH and it is a marker of late endosomes (Castellanos-Castro et al., 2016a). We investigated whether this phospholipid interacts with EhVps23 in the convoluted membranous structures observed through TEM (see **Figure 4**). In basal conditions, LBPA was found in vesicles in the cytoplasm and as discrete spots in the plasma membrane. Interestingly, 5 min after the RBCs stimulus was given; both molecules were visualized around the ingested RBCs and MVBs, although we also observed many clumps of LBPA without any interaction with EhVps23 (**Figures 7A, B**). Inside large phagosomes, networks of strands stained by the α-LBPA antibody appeared close to EhVps23 covering big areas of the phagosomes, and its ramifications reached the plasma membrane and the extracellular space (**Figure 7A**, 30 min). By TEM experiments, we also investigated whether LBPA was present in the convoluted membranous structures, co-localizing with EhVps23, using gold labeled antibodies of different sizes. TEM images showed both molecules together in the membranous structures (**Figure 7C** a-d), in vacuoles, and around phagosomes (**Figure 7C**). These results together give a panoramic of the complex process of phagocytosis, from the prey’s capture up to its digestion, and evidence distinct molecules and structures participating in these functions, as other authors have also shown (García-Rivera et al., 1999; Petri et al., 2002; Perdomo et al., 2016; Javier-Reyna et al., 2019). They also evidenced the role of EhADH, ESCRT proteins and LBPA in phagocytosis.

### Molecular Docking and Immunoprecipitation Assays Confirm EhVps23 Binding to EhUbiquitin, EhADH, LBPA and EhVps32

**Figure 8:**
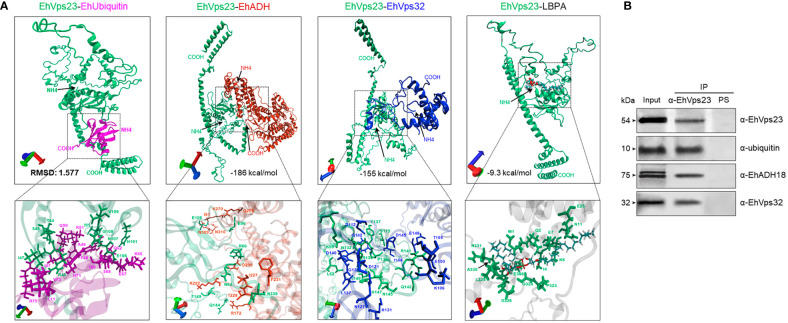
Molecular docking of EhVps23 with EhUbiquitin, EhADH, EhVps32 and LBPA and immunoprecipitation assays. **(A)** Three‐dimensional conformation of proteins and association of the EhVps23 (green)/Eh-Ubiquitin (pink), EhVp23 (green)/EhADH (red), EhVps23 (green)/EhVps32 (blue) and EhVps23 (green)/LBPA. Lower panels: magnification of the residues that allow the interaction between the pairs of molecules. **(B)** Immunoprecipitation. HM1-trophozoites in basal conditions were lysed and immunoprecipitated using the α-EhVps23 antibody or pre‐immune sera (PS). Immunoprecipitated proteins were analyzed by western blot using α-EhVps23, α-EhADH18, α-ubiquitin, and α-EhVps32 antibodies. Numbers at left: proteins molecular weight.

We delve into the study of EhVps23 putative interactions with EhUbiquitin, EhADH, EhVps32 and LBPA by docking analysis and immunoprecipitation assays. First, we aligned the homologous crystal structure of the complex Vps23-ubiquitn (from *S. cerevisiae)* (1UZX), with the EhUbiquitin crystal (4GSW) (Teo et al., 2004; Bosch and Siderovski, 2013). Then, we used the coordinates from this analysis to carry out a second alignment with the EhVps23 3D model obtained here. The structural alignment presented an RMSD of 1.577 (**Figure S4**), whereas the interaction site between EhVps23 and EhUbiquitin matched with the binding site reported by the crystal structure of *S. cerevisiae* Vps23 interacting with *Bos taurus* ubiquitin (Teo et al., 2004). In docking analysis, the binding site between EhUbiquitin and the EhVps23 UEV domain appeared conserved (**Figure 8A**). The residues from EhVps23 binding to EhUbiquitin were: T44, S45, R46, I47, I91, H101, E106, N107, G108 and V109, while the residues from EhUbiquitin were: L11, I47, F48, A49, G50, K51, Q65, K66, E67, S68, T69, H71, V73 and R75 (**Figure 8A**).

Docking analysis also predicted that EhVps23 interacts with EhADH, EhVps32 and LBPA. The global free energy of EhVps23-EhADH interaction was calculated in -186.14 kcal/mol, given by two salt bridges and thirteen hydrogen bonds. The EhVps23 residues that interacted with EhADH were N64, R66, E96, E106, Q144, T149 and N339 located mainly in the UEV domain; meanwhile, in EhADH were R172, D219, K222, I227, T228, F231, Q278, K270, N309 and N310, located in the Bro1 domain (**Figure 8A**). The EhVps23-EhVps32 interaction showed a global free energy of -155.13 kcal/mol. The binding site was formed by four salt bridges and fifteen hydrogen bonds. The residues from EhVps23 interacting with EhVps32 were L62, K99, N132, R136, Y137, P138, R141, Q142, N145, H147, T149 and S175 located mainly in the UEV domain. The residues from EhVps32 were T105, K106, D112, D121, N127, C131, L137, G138, E139, D140, L141, Q142, I144, D145, E148, and E150, located in the Snf7 domain (**Figure 8A**).

EhVps23 bound to LBPA with an energy of -9.3 kcal/mol. The residues from EhVps23 interacting with LBPA were M1, Q2, I4, E7, K8, N11, E25, P323, G324, D326, L329, A330, N331 and E386, located in amino terminus and between UEV and Vps23 core domains (**Figure 8A**).

These interactions were experimentally tested by immunoprecipitation assays using α-EhVps23 antibodies and total extracts of trophozoites. EhUbiquitin, EhADH and EhVps32 appeared in the immunoprecipitates. As a negative control we used a preinmune serum (**Figure 8B**). These results evidenced the direct or indirect association of EhVps23 with these molecules involved in the phagocytosis process.

### The Knock-Down of the *Ehvps23* Gene Diminishes the Rate of Phagocytosis and Migration of Trophozoites

**Figure 9:**
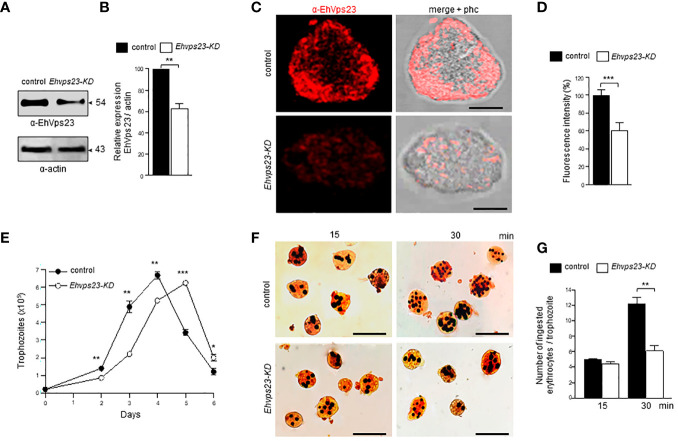
Silencing of the *Ehvps23* gene in *E*. *histolytica*. The *Ehvps23* gene was knocked down using the *pL4440Ehvps23_1-341_*_pb_ plasmid as described in materials and methods. **(A)** Western blot using lysates of non-silenced (control) and silenced (*Ehvps23*-KD) trophozoites in basal conditions, using the α-EhVps23 and the α-actin antibodies. **(B)** Densitometric analysis of bands showed in **(A)**, normalized against the actin protein bands. **(C)** Representative image of confocal microscopy of non-silenced and *Ehvps23*-KD trophozoites under basal condition using the α-EhVps23 antibody, and a TRITC-secondary antibody. Scale bar = 10 μm. **(D)** Fluorescence intensity measured by pixels. **(E)** Growth curve of *Ehvps23*-KD. **(F)** Novikoff staining of trophozoites after erythrophagocytosis. **(G)** Quantification of ingested erythrocytes by the trophozoites. Data represent the mean and standard error of the erythrocytes number counted inside of 100 trophozoites. *P < 0.05, ** P < 0.01, ***P < 0.001.

To obtain additional data on the function of EhVps23, we knocked-down the gene using dsRNA (Solis et al., 2009). Western blot assays using silenced trophozoites (*Ehvps23*-KD), evidenced that EhVps23 is expressed between 35 to 40% less in the knocked down trophozoites than in the control (**Figures 9A, B**). The immunofluorescence assays corroborated these results (**Figures 9C, D**). The rate of growth of the *Ehvps23*-KD trophozoites was retarded, but after five days of incubation at 37°C, cultures reached the same number of cells that the control did at four days (**Figure 9E**). These results are inconcordance with results obtained in other systems (Leung et al., 2008).

Next we evaluated the role of EhVps23 in the rate of phagocytosis using the silenced trophozites. Interestingly, at 15 min, the rate of phagocytosis of the *Ehvps23*-KD trophozoites was like the controls, but at 30 min, the *Ehvps23*-KD cells phagocytosed half of the RBCs than the control cells (**Figures 9F, G**). These results strengthen the assumption that the EhVps23 protein is involved in the phagocytosis of trophozoites, probably due to its active participation in vesicular trafficking.

## Discussion

**Figure 10:**
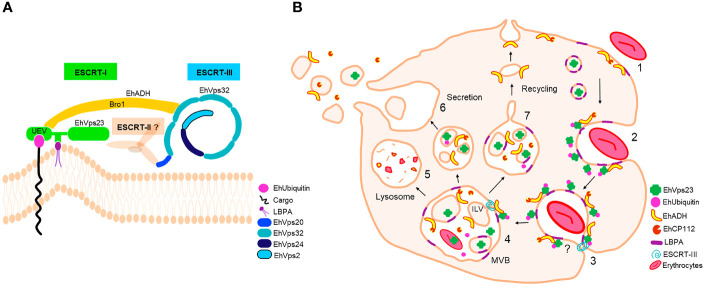
ESCRT machinery of *E*. *histolytica* and EhVps23 participation. **(A)** ESCRT machinery identified in *E*. *histolytica*. EhVps23 member of ESCRT-I complex interacts with LBPA, during cell membranes binding. The UEV domain of EhVps23 interacts with ubiquitinated proteins and EhADH. EhADH recruits to EhVps32 (ESCRT-III complex). EhVps20, EhVps32, EhVps24 and EhVps2 (ESCRT-III) polymerize generating helical structures to generate ILVs. **(B)** EhVps23 in phagocytosis. (1) Attachment to human erythrocytes to the trophozoites. (2) Phagocytic cup formation with the presence of EhVps23. (3) EhVps23 surrounds the nascent phagosome. (4) MVBs formation is triggered by the action of ESCRT-III polymers and EhVps23. (5) MVBs fuse to lysosomes or (6) to the cytoplasmic membrane to expel the ILVs content. (7) Pathways that direct proteins to the plasma membrane through recycling vesicles.

We have studied the ESCRT-I complex here, particularly the participation of its EhVps23 protein, the only one that we have detected in phagocytosis and vesicular trafficking in *E. histolytica*. Although drugs are available to combat this parasite, it continues causing 100 million infections and 100 thousand deaths each year (Mortimer and Chadee, 2010). The relevance of this work relies in the following facts: i) It provides new evidence on the concerted participation of several molecules with EhVps23 in vesicular trafficking and phagocytosis of this parasite (**Figure 10A**). ii) Phagocytosis is a pivotal event in the aggressive mechanism of *E. histolytica* trophozoites during tissue invasion; the knowledge of proteins involved in this function will give clues to combat this parasitosis. iii) Vesicular trafficking is crucial during phagocytosis and tissue invasion, thus, a better knowledge of the molecules involved these events, will help to uncover molecular mechanisms that are performed during these functions. iv) The ESCRT machinery has been poorly investigated in protozoan parasites (Leung et al., 2008; Lopez-Reyes et al., 2010; Lumb et al., 2011; Bañuelos et al., 2012; Ali et al., 2014; Avalos-Padilla et al., 2015; Avalos-Padilla et al., 2018; Iriarte et al., 2018; Saha et al., 2018; Moyano et al., 2019). Furthermore, protozoa and plants, exhibit remarkable differences with other eukaryotes, hence, this research can provide novel data to understand the evolutionary process of the ESCRT machinery. Thus, the Vps23 protein was also found in other *Entamoeba* species, that appeared grouped in a single clade, remarking the common origin of the protein in these amoebae species (**Figure 1C**). v) By their function as the core of vesicular trafficking and their divergence with mammalian cells, the ESCRT proteins are good targets for developing vaccines and therapeutic methods against pathogens. In fact, the role of the ESCRT proteins in virulence has been already reported for *Cryptococcus neoformans* (Hu et al., 2013) and *Candida albicans* (Dawson et al., 2020), among others.

We only found EhVps23 as a member of ESCRT-I and we did not find the canonical proteins forming the ESCRT-0. However, these findings did not discard the presence of orthologues with high divergence in the parasite. Instead, other molecules could perform the tasks carried out by ESCRT-0, such as Tom1 and others can interact with EhVps23 to form the ESCRT-I complex, however, we do not know this. In *Arabidopsis* and *D. discoideum* (Blanc et al., 2009; Mosesso et al., 2019), Tom1 recognizes ubiquitinated cargo and gathers the proteins that will be conducted through ESCRT-I to the MVBs. In fact, Tom1 is present in *E. histolytica* (Herman et al., 2011) and its functions are currently under study in our laboratory.

Confocal and TEM observations evidenced that in trophozoites in basal state, EhVps23 is in vesicles (**Figure 4**), like in *T. brucei* (Leung et al., 2008; Silverman et al., 2013). During phagocytosis, it moves to the site of contact of trophozoites with RBCs, interacting with other molecules that may be acting as receptors for the target cell. Then, the prey and these molecules are internalized and transported through endosomes to phagosomes and MVBs where ILVs are formed (**Figures 3**, **4**). From there, proteins may be conducted for recycling, degradation, or secretion (**Figures 10A, B**). EhVps23 is present along the event with the participation of membranous structures, which appeared under the microscope as round and elongated vesicles that connect to each other and were marked by α-EhVps23 and α-LBPA antibodies. This active vesicular trafficking involved the participation of EhVps23, EhADH and LBPA and digested hemoglobin appeared in erythrocytes-containing phagosomes.

Our work give evidence of a significant number of molecules involved in phagocytosis, that interact with EhVps23 along the vesicular traffick and phagocytosis. Immunofluorescence and immunoprecipitation as well as molecular docking analysis predicted that EhVps23 binds to EhUbiquitin, EhADH and EhVps32 through the UEV domain (**Figure 10A**). The binding to ubiquitin could be due to the direct ubiquitination of EhVps23, because the protein has lysines capable of receiving this modification. But it is also possible that the UVE domain of EhVps23 allows it to interact with ubiquitinated proteins, or both speculations could be true. The interaction seems to be similar to those reported for the binding of ScVps23-BtUb (Teo et al., 2004) and HsTSG101-PTAP-containing proteins (Im et al., 2010). On the other hand, the interaction between LBPA and EhVps23 was predicted to be carried out through amino acids located between the UEV and Vps23 core domains. This is the first report on binding of EhVps23 to LBPA (Urbé et al., 2000; Raiborg et al., 2001).

In conclusion: i) Our work give a panoramic of the complex interactions among distinct molecules, including proteins and LBPA, during the vesicular trafficking and phagocytosis of *E. histolytica*. ii) The EhVps23 protein, the only member of the ESCRT-I complex detected here, is a pivotal molecule in both functions in trophozoites: two indispensable functions for the parasite survival and virulence expression. iii) There is an intimate relationship among EhADH, EhVps32, EhVps23, EhUbiquitin and LBPA to perform phagocytosis, together with other molecular mechanisms and proteins involved in these functions, including the calcium binding proteins (Babuta et al., 2020; Kumar et al., 2020), Rab proteins (Javier-Reyna et al., 2019; Verma et al., 2020), as well as the Gal/GalNac (Petri et al., 2002), KERP1 protein (Perdomo et al., 2016), the cytoskeleton (Rath and Gourinath, 2020) and some others. iv) The distinct strategies and experiments performed here, evidenced the presence of abundant vesicular structures, tubules, and networks that arise, interact, and disappear during phagocytosis. Thus, the primitive protozoan *E. histolytica* is an excellent model to study vesicular trafficking, as well as the role of the ESCRT complex and its proteins; and by their differences with other organisms, it is also a suitable model for evolutionary studies to understand the nature, and phylogenetic relationships among the ESCRT machinery in other species.

## Data Availability Statement

Most contributions are included in the article. Other raw data supporting this research will be made available by the authors, without undue reservation. Further inquiries can be directed to the corresponding author.

## Author Contributions

AG, research, methodology, writing. RJ-R, research, methodology, supervision, and writing. GG-R, research methodology and supervision. CB, supervision and writing. SM, JO-L, BC-M, and LS-V, methodology. EO, supervision, writing, and research. All authors contributed to the article and approved the submitted version.

## Funding

This work was supported by the National Council for Science and Technology (Conacyt) of Mexico (grant A1-S8380 for EO), and RJR received a Conacyt Postdoctoral Fellowship.

## Conflict of Interest

The authors declare that the research was conducted in the absence of any commercial or financial relationships that could be construed as a potential conflict of interest.

## Publisher’s Note

All claims expressed in this article are solely those of the authors and do not necessarily represent those of their affiliated organizations, or those of the publisher, the editors and the reviewers. Any product that may be evaluated in this article, or claim that may be made by its manufacturer, is not guaranteed or endorsed by the publisher.
