# Supplementary figures and images for: EhVps23: A Component of ESCRT-I That Participates in Vesicular Trafficking and Phagocytosis of Entamoeba histolytica
Source: Front Cell Infect Microbiol. 2021 Oct 29;11:770759. doi: 10.3389/fcimb.2021.770759 (PMC8588831; doi:10.3389/fcimb.2021.770759)

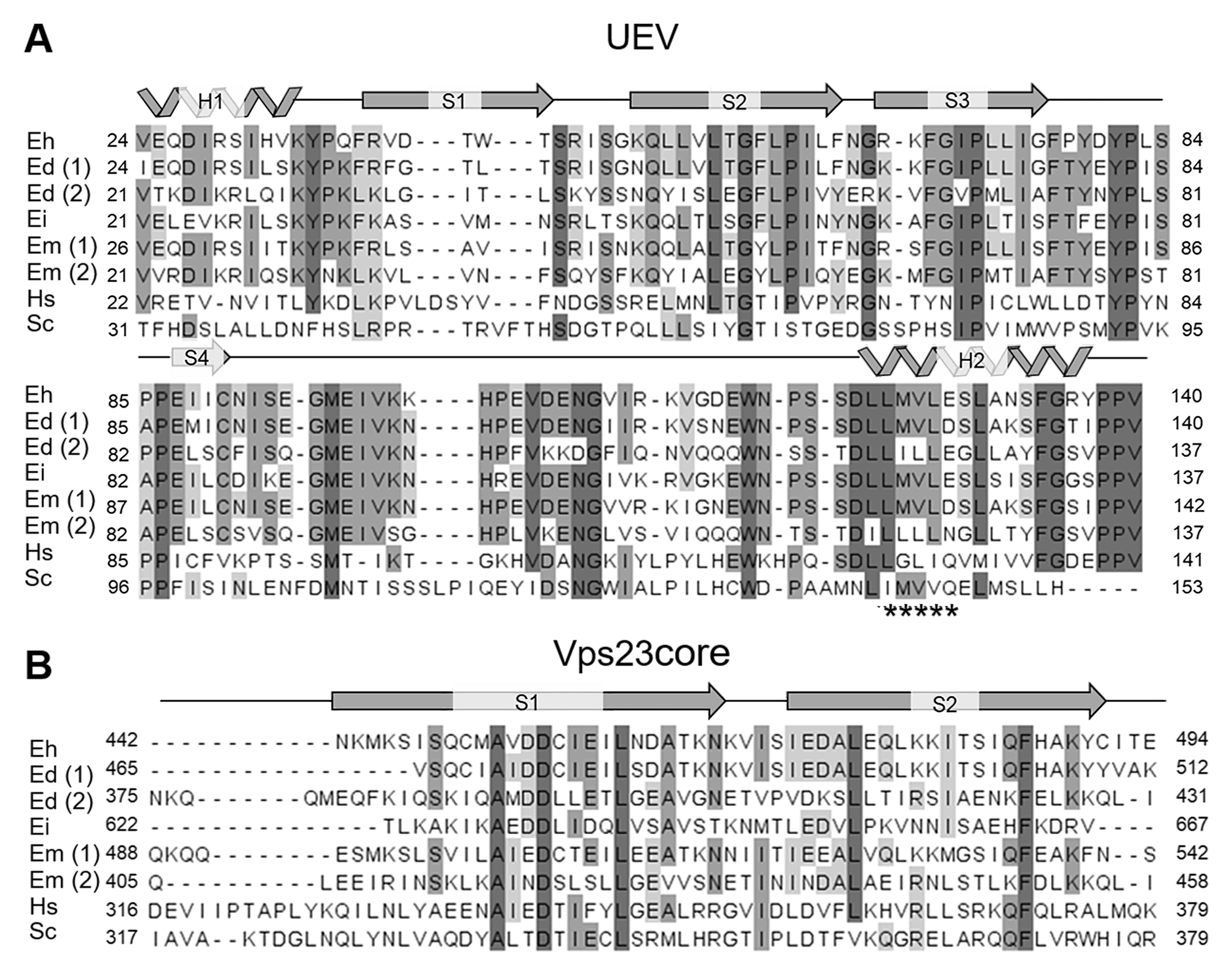

Supplement: Supplementary file 1 [file Image_1.tif]

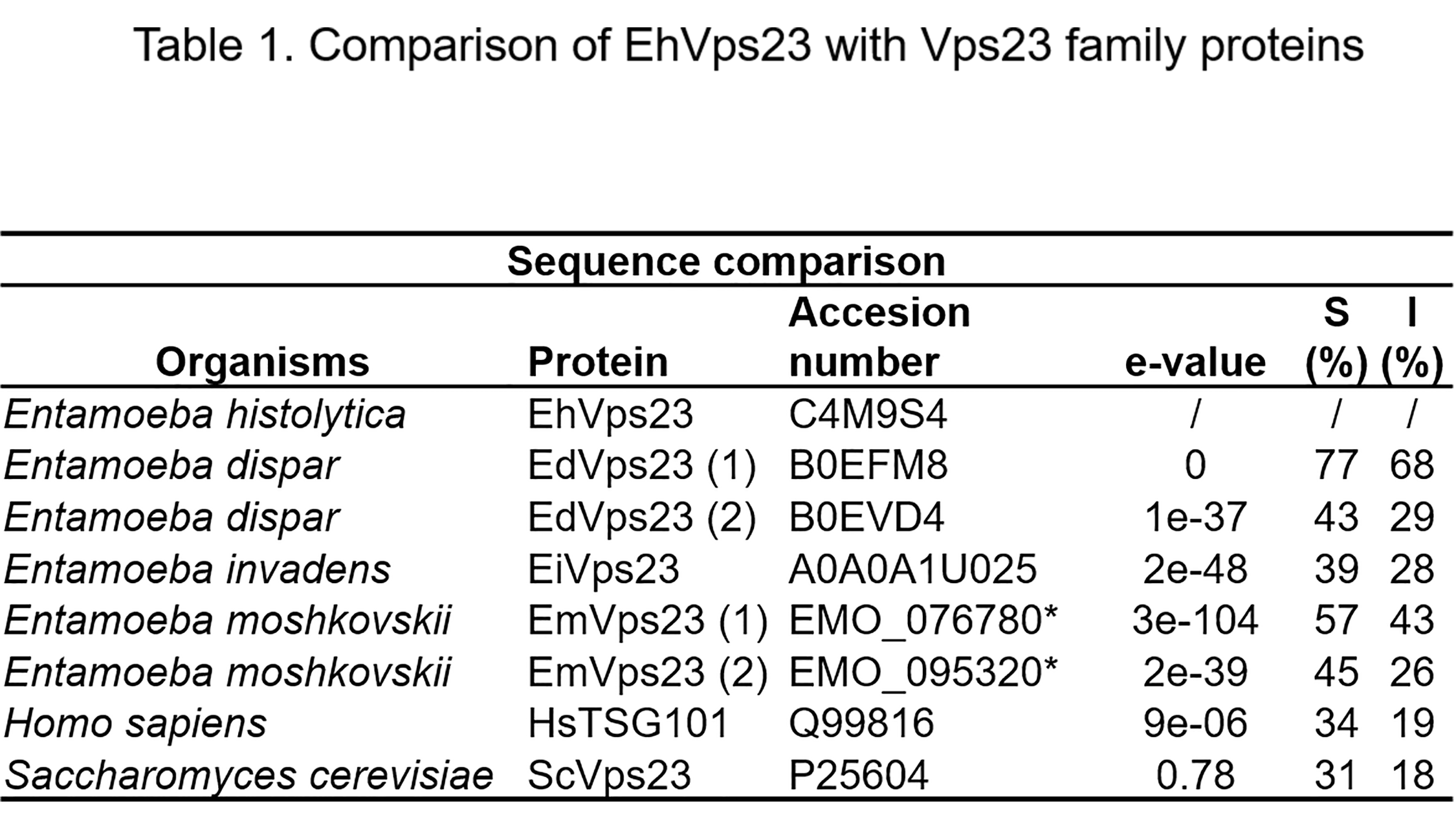

Supplement: Supplementary file 2 [file Image_2.tif]

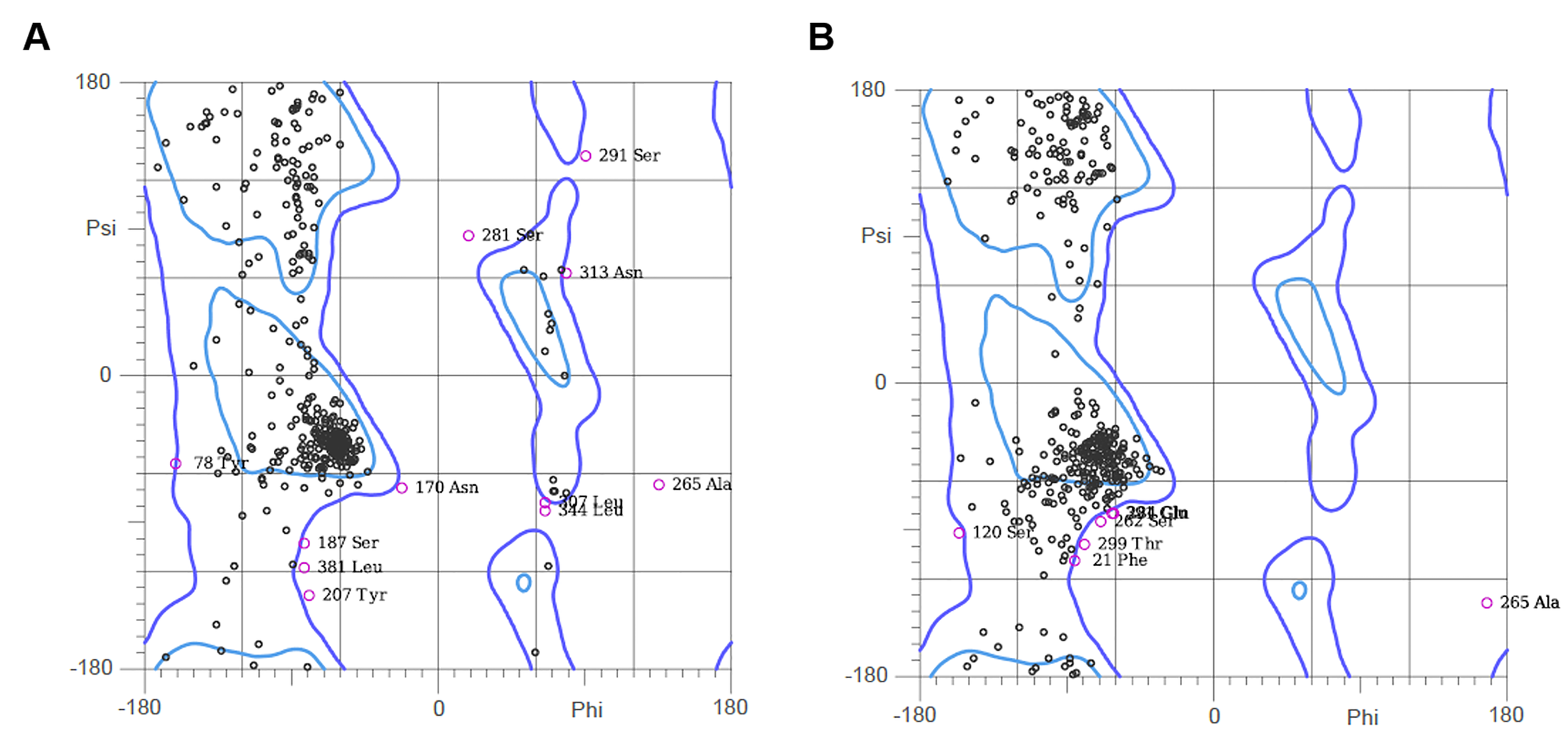

Supplement: Supplementary file 3 [file Image_3.tif]

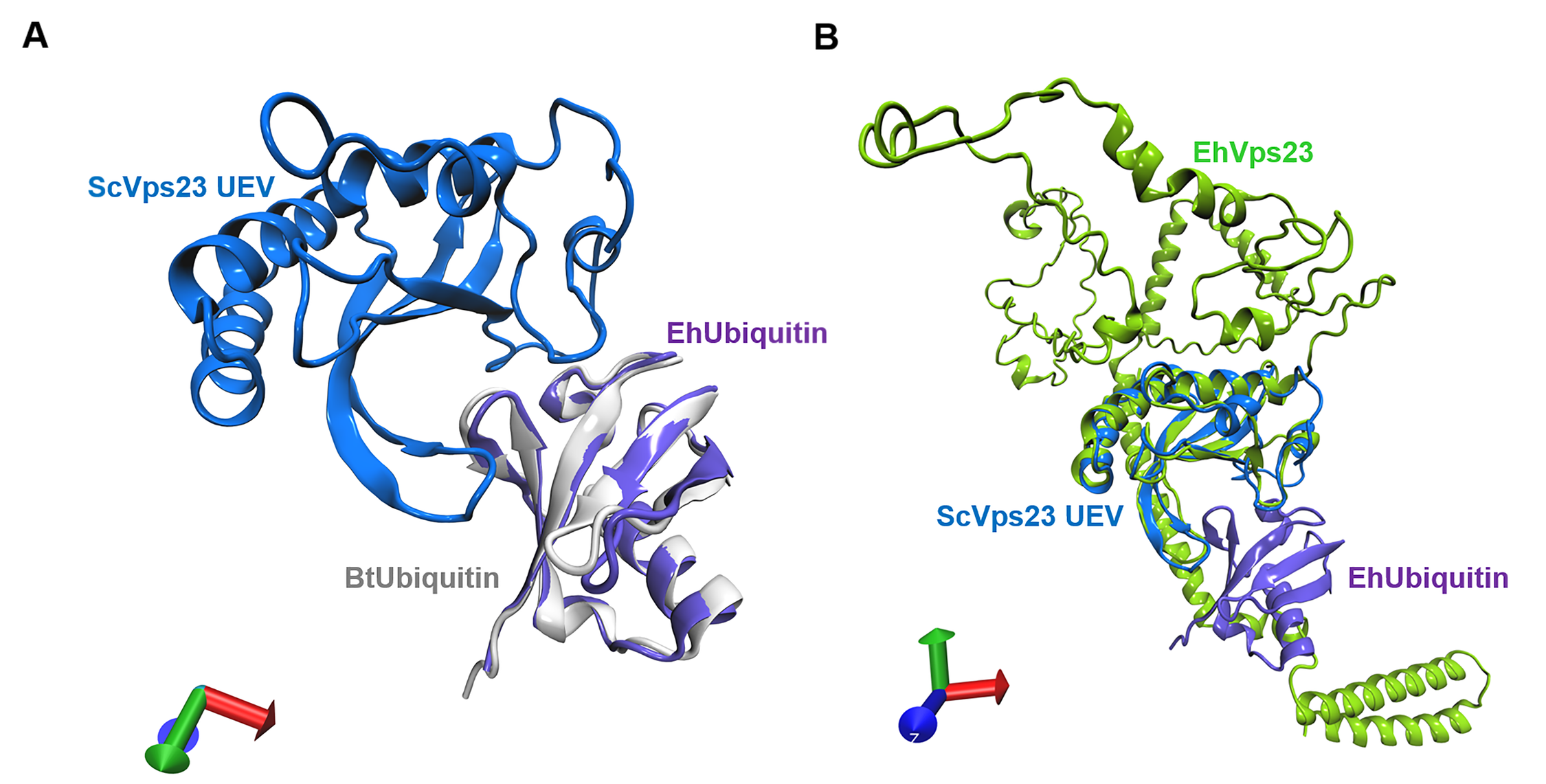

Supplement: Supplementary file 4 [file Image_4.tif]
